# The Bone-Protecting Efficiency of Chinese Medicines Compared With Western Medicines in Rheumatoid Arthritis: A Systematic Review and Meta-Analysis of Comparative Studies

**DOI:** 10.3389/fphar.2018.00914

**Published:** 2018-08-30

**Authors:** Xiao Cai, Xiu-Min Chen, Xuan Xia, Kun Bao, Rong-Rong Wang, Jian-Hong Peng, Hai-Jun Liu, Qiao-Wen Yang, Jing-Yao Yan, Mao-Jie Wang, Hua Yu, Jin-Jian Lu, Yuan-Jia Hu, Per-Johan Jakobsson, Ze-Huai Wen, Run-Yue Huang, Qing-Chun Huang

**Affiliations:** ^1^The Second Clinical College, Guangzhou University of Chinese Medicine, Guangdong Provincial Hospital of Chinese Medicine, Guangzhou, China; ^2^Department of Rheumatology, Dongguan Hospital of Traditional Chinese Medicine, Dongguan, China; ^3^Department of Rheumatology, Panyu District Central Hospital, Guangzhou, China; ^4^University Medicial Center Utrecht, Utrecht, Netherlands; ^5^State Key Laboratory of Quality Research in Chinese Medicine, Institute of Chinese Medical Sciences, University of Macau, Taipa, Macau; ^6^Rheumatology Unit, Department of Medicine, Karolinska Institutet and Rheumatology Clinic, Karolinska University Hospital Solna, Stockholm, Sweden; ^7^Key Unit of Methodology in Clinical Research, The Second Affiliated Hospital, Guangzhou University of Chinese Medicine, Guangdong Provincial Hospital of Chinese Medicine, Guangzhou, China; ^8^Guangdong Provincial Key Laboratory of Clinical Research on Traditional Chinese Medicine Syndrome, Guangzhou, China

**Keywords:** Rheumatoid Arthritis (RA), Chinese medicine, bone-protecting efficiency, systematic review, meta-analysis

## Abstract

**Background:** Rheumatoid Arthritis (RA) is a systemic autoimmune disease leading to joint destruction. The prevention of bone and cartilage destruction has received increased attention in recent years.

**Objective:** To evaluate the current evidences regarding the bone-protecting efficacy of Chinese medicine or the combination of Chinese medicine and Western medicine for RA.

**Methods:** We comprehensively searched PubMed, Embase, the Cochrane Library (www.thecochranelibrary.com), the China National Knowledge Infrastructure (CNKI), the Database for Chinese Technical Periodicals (VIP), and SinoMed. We then performed a systematic review and cumulative meta-analysis of all randomized controlled trials (RCTs) assessing the two therapy methods.

**Results:** Sixteen studies including 1,171 patients were included in the final analysis. The results showed that Chinese medicine could significantly improve the bone mineral density (BMD) (mean difference [MD] = 0.05 /g·cm^−2^, 95% CI [0.03, 0.08], *P* < 0.00001), and decrease the serum matrix metalloproteinase 3 (MMP-3) ([SMD] = −2.84, 95% CI [−4.22, −1.47], *P* < 0.0001).

**Conclusions:** Chinese medicine may provide an efficiently alternative choice for the treatment of RA in terms of the bone-protecting efficiency. Given the inherent limitations of the included studies, future well-designed RCTs are required to confirm and update the findings of this analysis.

## Introduction

Rheumatoid Arthritis (RA) is a chronic systemic autoimmune disease with symmetric inflammation of aggressive multiple joints (Miossec, 2013). As the most common inflammatory rheumatic disease, the prevalence of RA is about 0.5–1.0% in the world (Tanaka et al., 2014). In China, up to 5 million people suffer from RA with an estimated prevalence of 0.34% (Zhang et al., 2013). The inflammatory cell infiltration of synovium, pannus formation, and the progressive destruction of articular cartilage and bone destruction are the main pathological properties of RA (Mcinnes and Schett, 2011). The data from epidemiological investigations shows that about 90% of RA patients developed bone erosions within 2 years of the disease onset, eventually leading to joint deformities or even disability (Miossec, 2013; Nam et al., 2014). Therefore, RA brings with it a heavy burden and great pain to the families, patients, and even the society as a whole. The question of how to prevent the bone and cartilage from irreversible destruction thus becomes a key issue that doctors and scientists are currently paying more attention to and strive to resolve.

Up to date, there are no systematic reviews and meta-analysis regarding bone-protecting efficiency about Chinese Medicine alone or in combination with Western Medicine in the treatment of RA. We therefore systematically searched and analyzed the available literature to evaluate the efficacy and potential advantages of Chinese Medicine (or a combination of Chinese and Western medicine), when compared with Western Medicine.

## Methods

### Criteria for considering studies for this review

#### Types of studies

All randomized controlled clinical trials.

#### Type of participants

Adults (usually over 18 years of age) with a diagnosis of RA either using the 1987 American College of Rheumatology (ACR) classification criteria (Arnett et al., 1988) for RA, or using the 2010 ACR/ European League Against Rheumatism (EULAR) classification criteria (Aletaha et al., 2010) for RA.

#### Type of interventions

All experimental groups were treated with oral Chinese medicine or combined Chinese and western medicine. The comparison arm was treated only with oral Western medicine.

#### Type of outcome measures

##### Primary outcomes

The evaluation of radiographic progression of hands. The classification standards refer to AHA standards (Arnett et al., 1988).

##### Secondary outcomes

The secondary outcomes include the bone mineral density (BMD) and the levels of serum matrix metalloproteinase 3 (MMP-3), a biomarker for bone destruction (Klimiuk et al., 2002).

### Search methods for identification of studies

We developed our search strategies sequentially. The following MeSH terms and their combinations were used to search in [Title/Abstract]: Rheumatoid Arthritis, random, control, and bone. We searched these terms in all databases in order to fit the requirements of the specific database style.

### Search strategies for identification of studies

We searched the following electronic databases.

1. PubMed, inception to 31 December 2017;
2. EMBASE, inception to 31 December 2017;
3. The Cochrane Library (www.thecochranelibrary.com), inception to 31 December 2017;
4. The China National Knowledge Infrastructure (CNKI), inception to 31 December 2017;
5. The Database for Chinese Technical Periodicals (VIP), inception to 31 December 2017;
6. SinoMed, inception to 31 December 2017.

In addition, we manually searched the reference lists of the included studies and previous review papers to find additional studies. All references were imported to an EndNote (x6) library and tagged with the name of the database.

### Data collection and analysis

#### Selection of studies

Two review authors assessed the titles and abstracts for all the records identified through the search strategies, retrieving full texts for all those that appeared to satisfy the following criteria: the type of study; type of participants; type of intervention; type of measurements. Data from the included studies were extracted and summarized independently by at least two of the authors. Any disagreement was resolved by a discussion among all the authors.

#### Data extraction and management

For data extraction, the review team allocated papers to different authors according to their areas of expertise, and two reviewers independently retrieved the details for each publication and tabulated them in a standardized form. The retrieved details include intervention (including characteristics and duration), assignment to groups (including the form of a drug, concealment, and comparability of groups), outcome measures, timing of measurements, adherence to intervention/control, sample size statistical analysis methods as well as adverse events and withdrawals. Two review (XC, RYH) authors independently extracted data from the reviews using a predefined data extraction form created as a Microsoft Excel® spreadsheet.

#### Assessment of risk of bias in included studies

Studies were rated for evidence level according to the criteria given by the Centre for Evidence-Based Medicines in Oxford, UK (Phillips et al., 2012). Assessment of risk of bias was undertaken for each included study using the Cochrane Collaboration's risk of bias assessment tool (Higgins and Green, 2008). Seven key domains were assessed by two review authors, which include sequence generation, allocation concealment, blinding of participants and personnel, blinding of outcome assessment, incomplete outcome data, selective outcome reporting, and other sources of bias. Pairs of review authors judged the key domains as “high risk,” “low risk,” or “unclear” risk of bias. In cases of disagreement between the review authors, the decision was made by consensus.

#### Measures of treatment effect

For each trial, data analysis employed a standard meta-analysis using the methods for continuous data. Mean differences (MD), standardized mean differences (SMD), and 95% confidence intervals (CIs) were calculated for the continuous outcomes (reporting mean and standard deviation of the mean). Where the standard deviations were not explicitly stated, we calculated them from the different means and their respective CIs or *P*-values.

#### Assessment of heterogeneity

Where appropriate, we formally assessed heterogeneity of the data using the *I*^2^ statistics (Higgins et al., 2003). We judged a value greater than 50% to represent substantial heterogeneity. If results were determined to be heterogeneous (that is *I*^2^ > 50%), a random-effects model would be used to further analyze the results. Where we detected this level of heterogeneity and there were sufficient studies available, we conducted subgroup analyses in an attempt to explain the heterogeneity.

#### Assessment of reporting biases

We used Egger's test to assess the possibility of publication bias with Stata 11.0.

### Data synthesis

#### Statistical analyses

Where there was no heterogeneity, we used a fixed-effect model, and where there was heterogeneity, we used a random-effects model if there was no clinical heterogeneity. The MD for pooled data in meta-analysis were calculated using a fixed model as outcomes were measured on the same standard scales. Otherwise, the SMD were calculated. Meta-analysis was facilitated by Review Manager 5.3 (Cochrane Collaboration, Oxford, UK) using the statistics as described below.

#### Subgroup analysis and investigation of heterogeneity

Where sufficient studies were available and the data was heterogeneous, we carried out separate meta-analyses for studies according to some factors including intervention duration, disease stage, and the form of traditional Chinese medicine (TCM).

#### Sensitivity analysis

In studies where calcium supplements were used, we planned the sensitivity analyses as a priority in order to explore the differences in effect size and to assess whether the conclusions were robust to the decision-making process. The sensitivity analyses included the following:

(1) The effect of risk of bias in included studies—defined as adequate allocation concealment and blinding of outcome assessors; (2) the effect of using calcium supplements.

## Results

### Description of studies

We undertook a comprehensive literature search, including screening of titles and abstracts. In total, we retrieved 25 fulltext references for further evaluation, including the manual searching of reference lists (9 further full-text studies).

**Figure 1:**
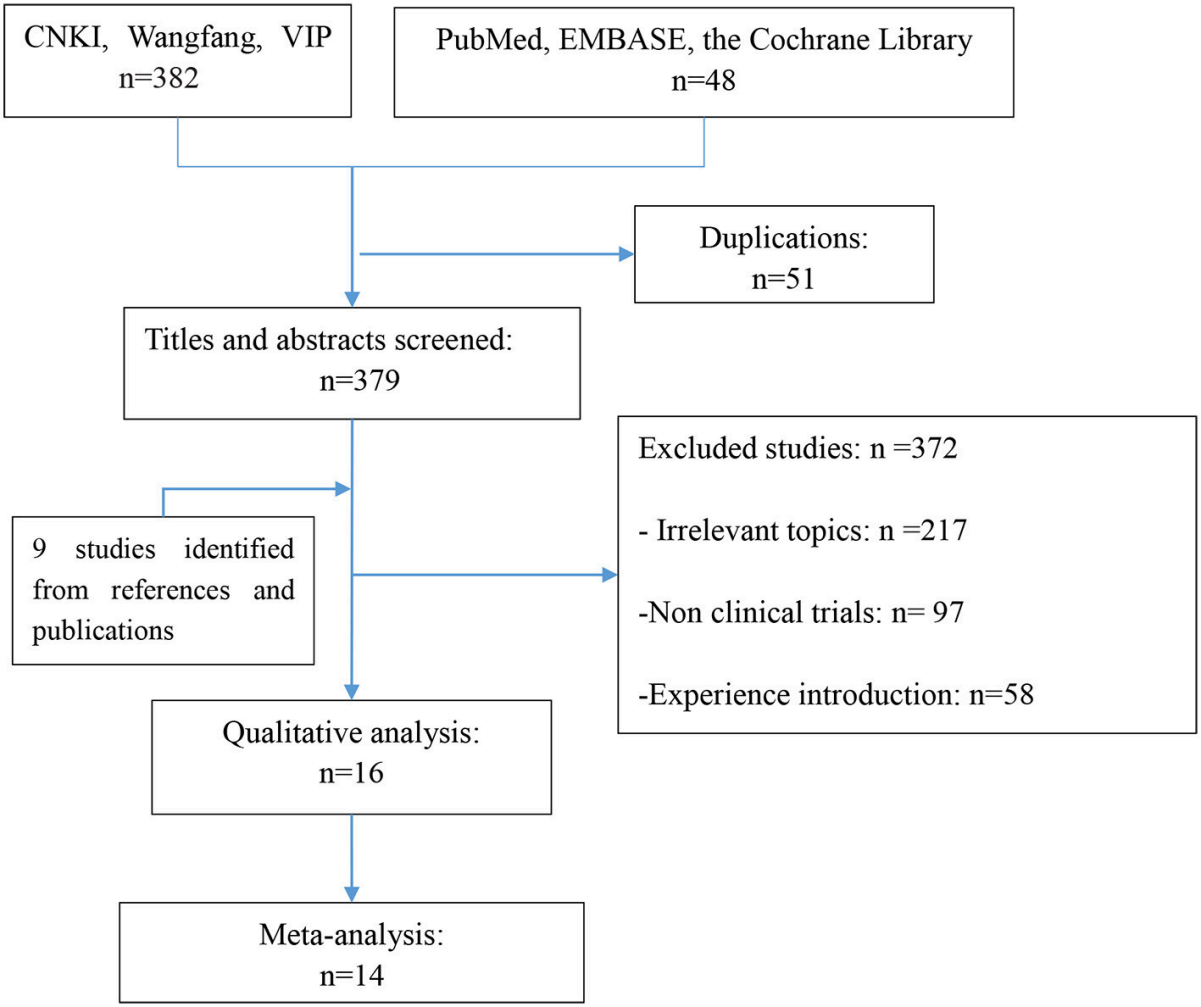
The study flow diagram.

Sixteen studies (Lu et al., 2002; Tang et al., 2005; Ma et al., 2009; Xue et al., 2009; Su, 2010; Li et al., 2012, 2017; Ouyang et al., 2013; He and Xiao, 2014; Ling et al., 2014; Liu, 2014; Liu et al., 2014; Pang et al., 2015; Wu et al., 2015; Xu et al., 2016; Zhang et al., 2016) including 1,171 cases (620 cases for oral Chinese medicine only or combined with western medicine, 551 cases for oral western medicine) fulfilled the predefined inclusion criteria and were included in the final analysis (Figure 1). Examination of the references listed for these studies and for the articles did not yield any further studies for evaluation. Agreement between the two reviewers was 90% for study selection and 92% for quality assessment of trials.

The study flow diagram and search summary are described in Figure 1 and Table 1, respectively.

**Table 1:** Characteristics of the included studies.

**Author**	**Year**	**Sample size**				**Disease stage (Y)**	**Age (Y)**	**Intervention methods**		**Outcomes**	**Intervention duration**	**Treatment of traditional Chinese medicine**
		**EG**	**(FP)**	**CG**	**(FP)**			**EG**	**CG**			
Lu Sijian	2002	40	(0.78)	20	(0.75)	2.67 ± 1.23	41.2 ± 11.8	CHD+MTX+SSZ	MTX+SSZ+Pacebo	Radiographic Progression	3 months	Clearing away heat and detoxifying, dispelling wind and dehumidification
Tang Tianfeng	2005	22	(0.77)	19	(0.79)	5.67 ± 3.66	49.61 ± 15	CHD	MTX+SSZ	MMP3	3 months	Clearing heat and detoxifying, activating blood and removing stasis
Xue Jing	2009	46	(0.74)	43	(0.93)	3.36 ± 6.08	47.14 ± 10.4	CPM_1_+MTX+NSAIds	MTX+NSAIds	MMP3	12 weeks	Eliminating swelling and relieving pain, activating blood and removing stasis
Ma Wukai	2009	32	(0.68)	31	(0.62)	4.57 ± 4.75	48.05 ± 14	CPM_2_+MTX+SSZ+NSAIDs	MTX+SSZ+NSAIds	Radiographic Progression	6 months	Warming meridian to relieve pain, activating blood and removing stasis
Su Linchong	2010	35	–	32	–	−	−	CHD+MTX+NSAIds	MTX+NSAIds	MMP3	3 months	Activating blood and removing stasis
Li Zhuoling	2012	27	(0.73)	28	(0.84)	1.07 ± 0.6	53.9 ± 10.85	CHD	CS+CCD	BMD	3 months	Invigorating kidney and removing stasis
Ouyang Guilin	2013	25	(0.68)	27	(0.67)	2.35 ± 1.27	35.24 ± 11.66	CPM_3_+MTX+NSAIDs	NSAIDs+MTX	BMD	6 months	Invigorating kidney and strengthening bone
He Dongchu	2014	30	(0.73)	30	(0.70)	0.45 ± 0.07	49.93 ± 11.89	CHD+MTX+LEF	MTX+LEF	MMP3	3 months	Dispelling wind and dispersing cold, dredging collaterals and relieving pain
Ling Yun	2014	48	(0.75)	48	(0.81)	9.75 ± 3.22	45.15 ± 4.71	CPM_4_+MTX+SSZ	MTX+SSZ	BMD	6 months	Dispelling wind and removing dampness, dredging collaterals and relieving pain
Liu Xiaodong	2014	72	–	35	–	7.2	66.4	CHD	NSAIDs	BMD	3.7–4.2 months	Differentiation and classification
Liu Zhuo	2014	29	(0.60)	29	(0.63)	3.74 ± 3.48	42.69 ± 17.96	CPM_5_+MTX+LEF	MTX+LEF+NSAIds	MMP3	2 months	Invigorating Qi, dispelling cold and dampness
Pang Xuefeng	2015	56	(0.73)	56	(0.77)	7.5 ± 2.44	43.65 ± 5.43	CHD+MTX+HCQ	MTX+HCQ	BMD	6 months	Nourishing liver and kidney, nourishing qi and blood
Wu Chunmei	2015	26	–	23	-	−	−	CPM_6_+MTX+LEF+NSAIDs	MTX+LEF+NSAIDs	MMP3	2 months	Activating blood to dispel cold, dispelling wind and dehumidification
Zhang Yanyan	2016	35	(0.89)	33	(0.91)	0.47 ± 0.14	42.53 ± 9.5	CHD+MTX+NSAIDs	MTX+NSAIDs	MMP3	24 weeks	Soothing the liver and regulating the spleen, dispelling wind and dehumidification
Xu Zejun	2016	30	(0.80)	30	(0.77)	4.56 ± 2.52	42.05 ± 2.79	CHD+CS	LEF+NSAIds+CS	MMP3	30 days	Tonifying the liver and kidney, dispelling wind and dehumidification
Li Jian	2017	67	(0.69)	67	(0.66)	3.3 ± 1.25	50.95 ± 5.97	CHD+MTX+NSAIds	MTX+NSAIds	MMP3	12 weeks	Removing cold and activating meridians, dispelling wind and dehumidification

*FP, Female proportion; SSZ, Sulfasalazine; MTX, Methotrexate; HCQ, Hydroxychloroquine; CHD, Chinese herbal decoction; CPM, Chinese patent medicines; NSAIDs, Non-steroidal anti-inflammatory drugs; CS, Calcium supplement; CPM1, Compound corypalis tablet; CPM2, Three Wu capsule; CPM3, Qianggu capsule; CPM4, Zheng Qingfeng Tongning tablet; CPM5, Gulao Yukang pill; CPM6, Qianggui Juanbi capsu*.

### Included studies

A total of 16 articles were included and all of these trials were performed in China. There were 11 articles (Lu et al., 2002; Tang et al., 2005; Ma et al., 2009; Xue et al., 2009; Su, 2010; Li et al., 2012; Ouyang et al., 2013; Ling et al., 2014; Liu et al., 2014; Pang et al., 2015; Xu et al., 2016) that used the ACR 1987 criteria and 4 studies (Liu, 2014; Wu et al., 2015; Zhang et al., 2016; Li et al., 2017) that used the 2010 revised criteria for the ACR/EULAR classification of RA.

The primary outcomes of the included studies were the evaluation of radiographic progression of hands. Only two studies (Lu et al., 2002; Ma et al., 2009) provided changes of X-ray image staging. In two studies (Lu et al., 2002; Ma et al., 2009) the data set was incomplete and, hence we were unable to conduct the analyses of their primary outcomes. Nine studies (Tang et al., 2005; Xue et al., 2009; Su, 2010; He and Xiao, 2014; Liu, 2014; Wu et al., 2015; Xu et al., 2016; Zhang et al., 2016; Li et al., 2017) including 626 patients provided the serum MMP-3 data. Five studies (Li et al., 2012; Ouyang et al., 2013; Ling et al., 2014; Liu et al., 2014; Pang et al., 2015) including 422 patients provided the BMD data. In most of the studies, the BMD was observed by using dual-energy x-ray absorptiometry (DEXA) and biological mechanics methods. Only one study used single-photon absorption to measure the BMD. In these studies, the BMD was assessed by testing the lumbar, vertebra, ulna, or undefined part.

### Excluded studies

Nine RCTs (two abstracts and seven articles) were excluded: two did not explain the diagnostic criteria, six did not include the outcomes of interest, and one used proprietary Chinese medicine in the control group.

### Risk of bias in included studies

**Figure 2:**
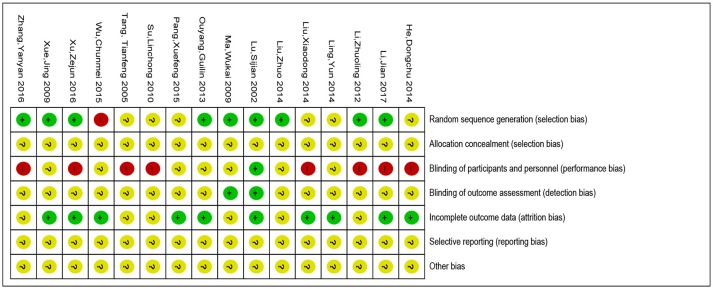
Risk of bias summary of included studies. “?”: unclear risk of bias; “–”: low risk of bias; “+”: high risk of bias.

**Figure 3:**
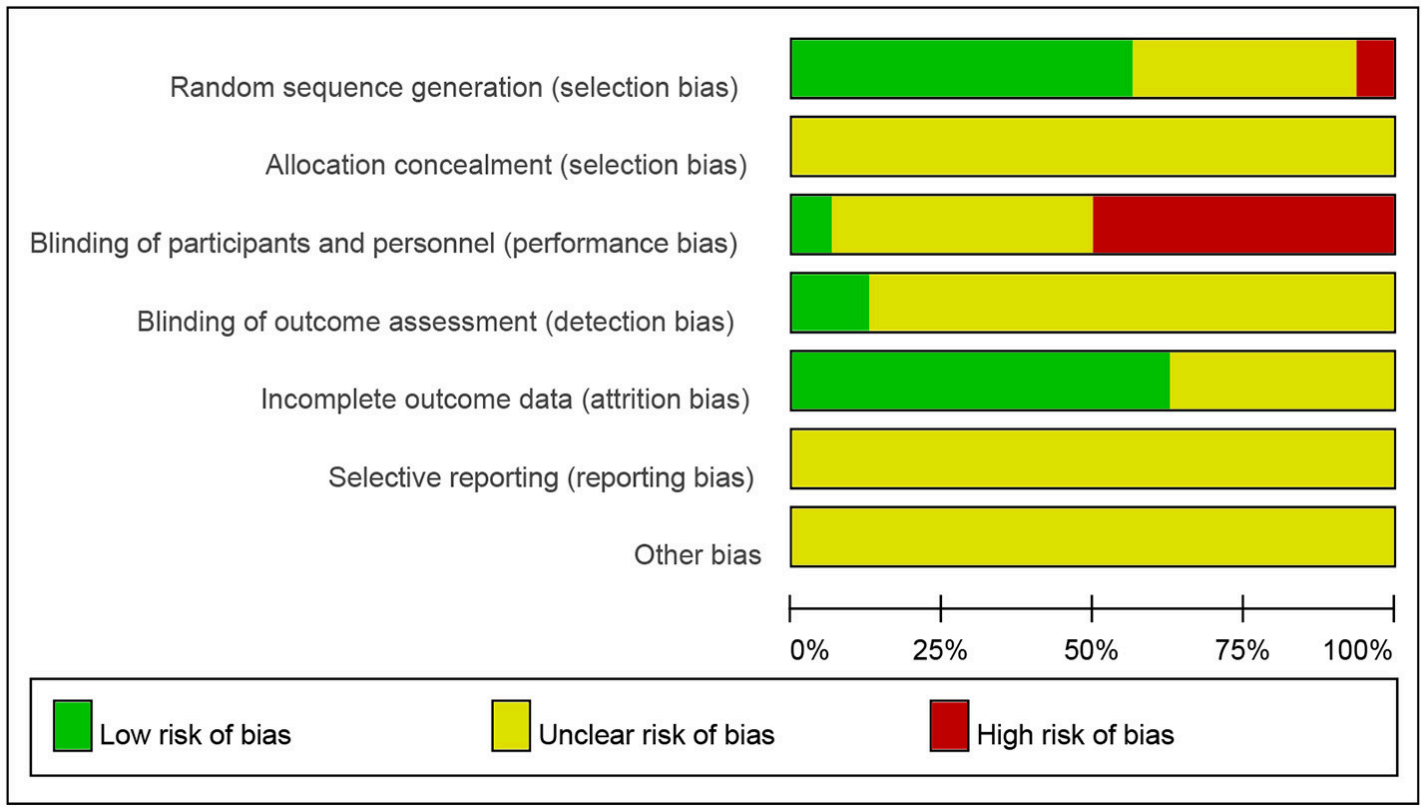
Evaluation for bias risk of included studies.

Overall, for most of the included studies the risk of bias was low or unclear. The methodological quality summary for each included study is presented in Figure 2. The review authors' judgments about each methodological quality item are presented as percentages across all the included studies in Figure 3.

### Allocation (selection bias)

Investigators described all the studies included as randomized controlled trials. Nine studies (Lu et al., 2002; Ma et al., 2009; Xue et al., 2009; Li et al., 2012, 2017; Ouyang et al., 2013; Liu, 2014; Xu et al., 2016; Zhang et al., 2016) adequately described the random method (low risk of bias). One study (Wu et al., 2015) reported a high risk of bias and the other studies did not clearly describe these methods (unclear risk of bias). Allocation concealment was assessed unclear in all the trials.

### Blinding (performance and detection bias)

Only one trial (Lu et al., 2002) was judged being at low risk of performance bias while eight were at high risk of bias. In 7 trials (Tang et al., 2005; Su, 2010; Li et al., 2012, 2017; He and Xiao, 2014; Liu et al., 2014; Xu et al., 2016; Zhang et al., 2016) the participants were not blinded and these trials were all judged as being at high risk of performance bias. We assessed low risk of detection bias in 2 trials (Lu et al., 2002; Ma et al., 2009) and unclear risk in 14 trials.

### Incomplete outcome data (attrition bias)

Ten studies (Lu et al., 2002; Xue et al., 2009; Ouyang et al., 2013; He and Xiao, 2014; Ling et al., 2014; Liu et al., 2014; Pang et al., 2015; Wu et al., 2015; Xu et al., 2016; Li et al., 2017) were judged being at low risk of attrition bias and others were judged as unclear risk.

### Selective reporting (reporting bias)

All trials were judged as unclear risk since the study protocols were not available and we did not have enough information in the study report to assess selective reporting.

### Other potential sources of bias

All trials were judged as unclear risk.

### Effects of interventions

Summary of the findings for the main comparison includes that Chinese medicine may provide an efficiently alternative choice for treatment of RA regarding its bone-protecting efficiency.

### Primary outcomes

#### Evaluation of radiographic progression of hands

Only two studies (Lu et al., 2002; Ma et al., 2009) provided changes of X-ray image staging, while two studies (Lu et al., 2002; Ma et al., 2009) did not provide complete data, which made it difficult to merge the analysis. In the Chinese Medicine groups the scores of radiographic progression improved significantly after treatment, whereas no significant difference was found in the Western Medicine groups after treatment.

### Secondary outcomes

#### Bone mineral density (BMD)

**Figure 4:**
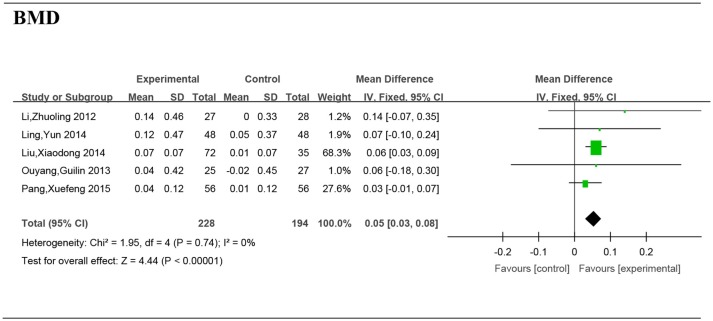
Forest plot and meta-analysis of bone mineral density. Experimental: the group of Chinese Medicine; Control: the group of Western medicine; RA, Rheumatoid Arthritis; SD, standard deviation; IV, inverse variance method; CI, confidence interval.

Pooling the data from 6 studies (Li et al., 2012; Ouyang et al., 2013; Ling et al., 2014; Liu et al., 2014; Pang et al., 2015) that measured the increase in bone mineral density in 422 patients, it was demonstrated that Chinese medicine (or combination of Chinese and western medicine) resulted in more significant effects than the western medicine groups (MD = 0.05/g·cm^−2^, 95 %CI [0.03, 0.08], *P* < 0.00001) (Figure 4).

#### The serum matrix metalloproteinase 3 (MMP-3)

**Figure 5:**
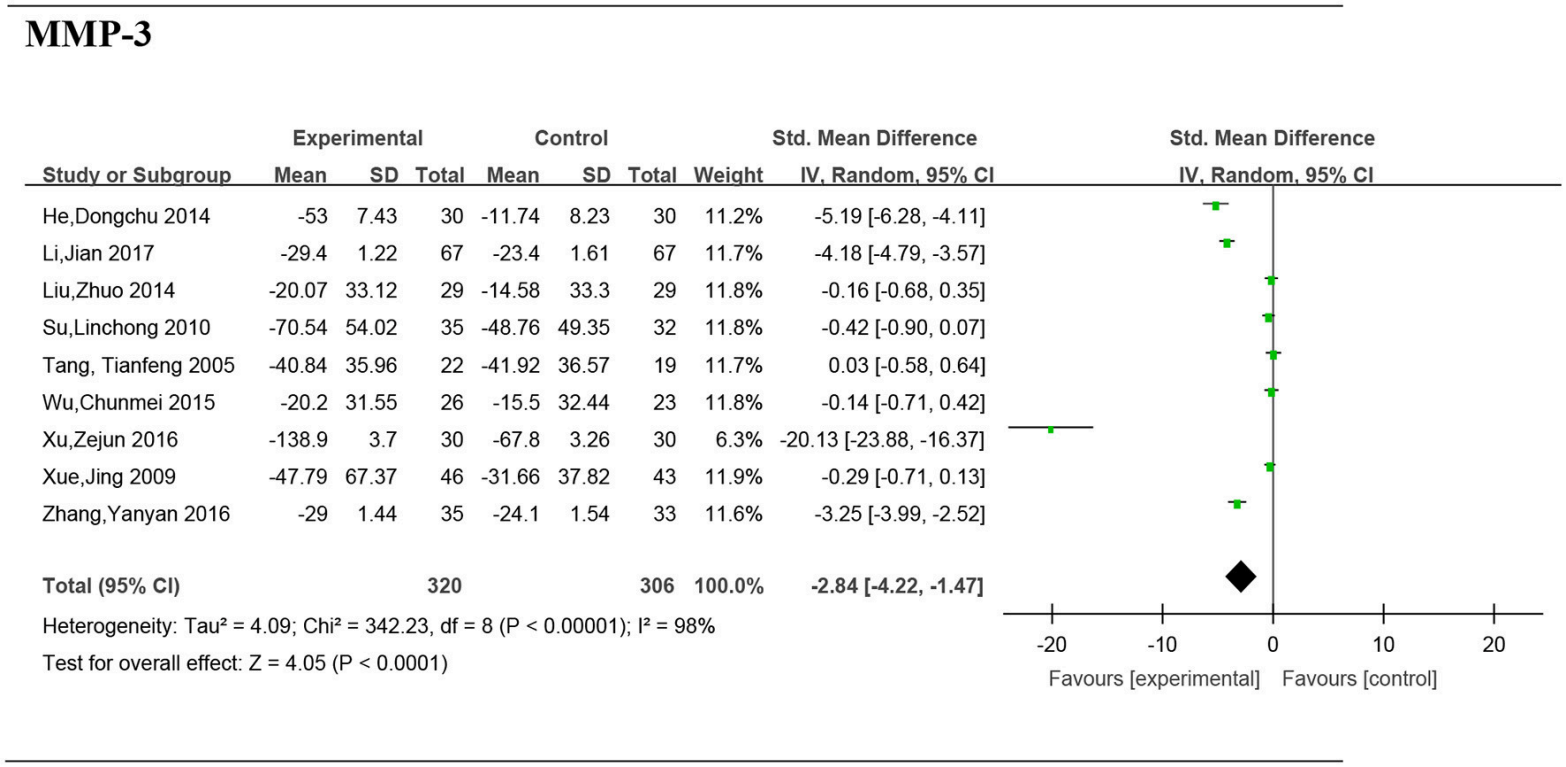
Forest plot and meta-analysis of the serum matrix metalloproteinase 3. Experimental: the group of Chinese Medicine; Control: the group of Western medicine; SD, standard deviation; IV, inverse variance method; CI, confidence interval.

Nine studies (Tang et al., 2005; Xue et al., 2009; Su, 2010; He and Xiao, 2014; Liu, 2014; Wu et al., 2015; Xu et al., 2016; Zhang et al., 2016; Li et al., 2017) including 626 patients provided the serum MMP-3 data. The *I*-squared was 98% and the *p*-value was <0.00001, so a random effects model was adopted for the meta-analysis. Significant differences were found in the reduction of serum MMP-3 levels between the Chinese medicine (or Chinese combined with Western medicine) group and Western medicine group (SMD = −2.84, 95%CI [−4.22, −1.47], *P* < 0.0001) (Figure 5).

### Subgroup analyses of the serum MMP-3 by intervention duration

#### Intervention duration−1 month

**Figure 6:**
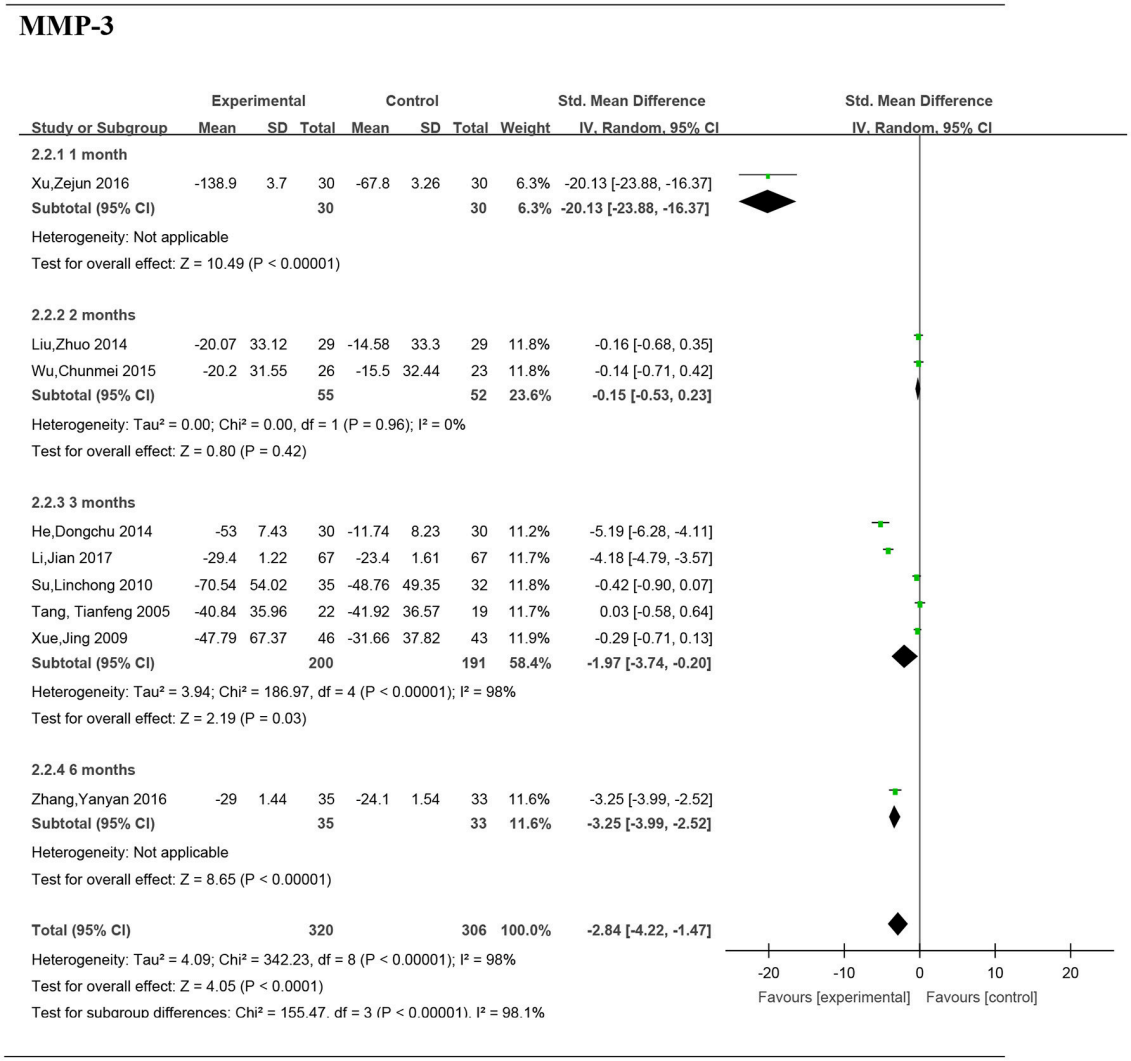
Forest plot and meta-analysis of the serum matrix metalloproteinase 3 of subgroup analysis by intervention duration. Experimental: the group of Chinese Medicine; Control: the group of Western medicine; SD, standard deviation; IV, inverse variance method; CI, confidence interval.

Data from one paper (Xu et al., 2016) reported the significant difference of serum MMP-3 between two groups (SMD: −20.13; 95% CI, −23.88 to −16.37; *P* < 0.00001) (Figure 6).

#### Intervention duration−2 months

Two studies (Liu, 2014; Wu et al., 2015) including 107 patients reported no significant difference of serum MMP-3 between the groups (SMD: −0.15; 95% CI, −0.5– 0.23; *P* = 0.42).

#### Intervention duration−3 months

Five studies (Tang et al., 2005; Xue et al., 2009; Su, 2010; He and Xiao, 2014; Li et al., 2017) including 391 patients reported a significant difference of MMP-3 between the groups (SMD: −1.97; 95% CI, −3.74 to −0.20; *P* = 0.03).

#### Intervention duration−6 months

Data from one paper (Zhang et al., 2016) reported a significant difference of serum MMP-3 between two groups (SMD: −2.84; 95% CI, −4.22 to −1.47; *P* < 0.00001).

### Subgroup analyses of the serum MMP-3 by disease stage

#### Disease stage, <1 year

Two studies (He and Xiao, 2014; Zhang et al., 2016) including 128 patients reported significant difference of MMP-3 between the groups (SMD: −4.18; 95% CI, −6.08 to −2.28; *P* < 0.0001).

#### Disease stage, >1 year

**Figure 7:**
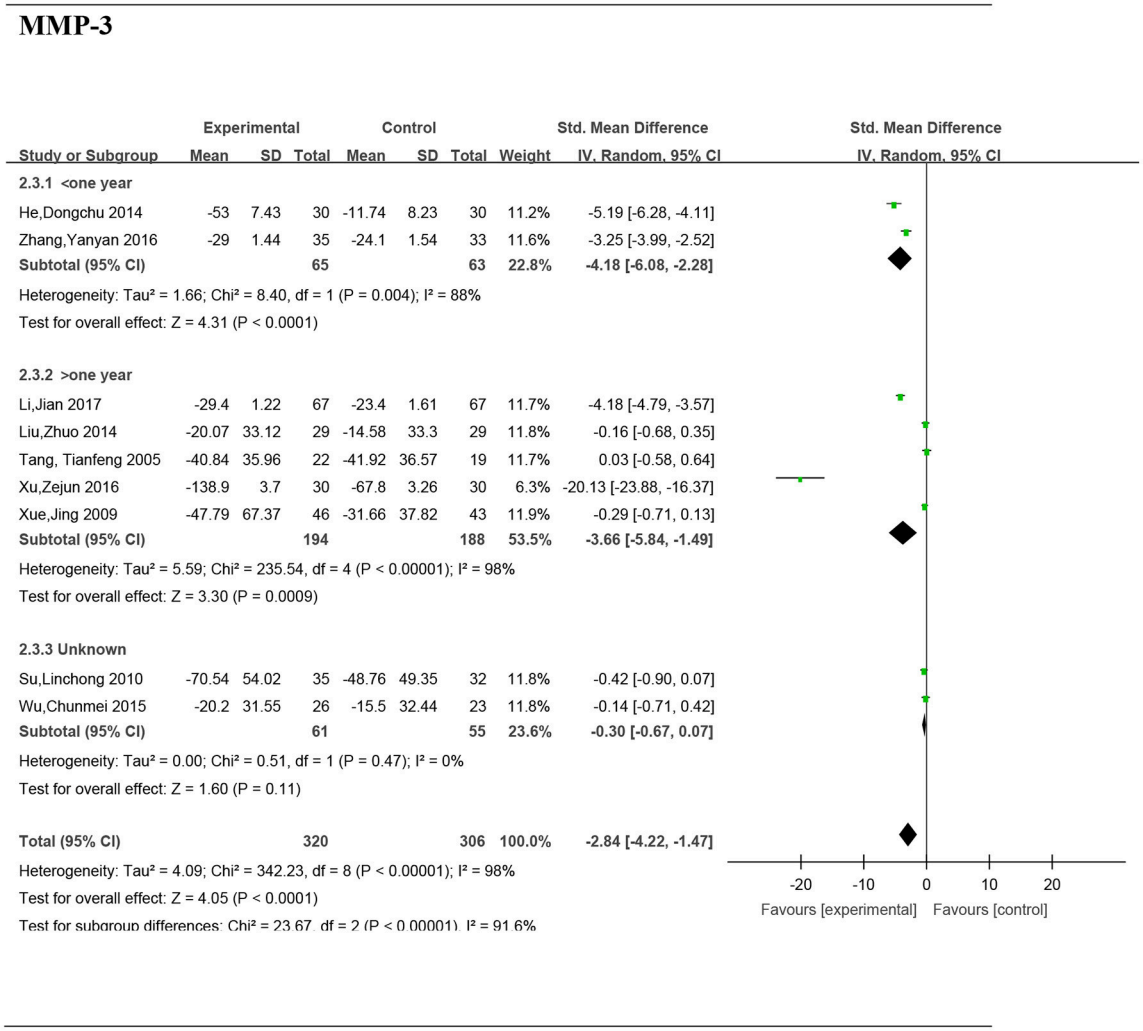
Forest plot and meta-analysis of the serum matrix metalloproteinase 3 of subgroup analysis by disease stage. Experimental: the group of Chinese Medicine; Control: the group of Western medicine; SD, standard deviation; IV, inverse variance method; CI, confidence interval.

Five studies (Tang et al., 2005; Xue et al., 2009; Liu, 2014; Xu et al., 2016; Li et al., 2017) including 382 patients reported a significant difference of MMP-3 between the groups (SMD: −3.66; 95% CI, −5.84 to −1.49; *P* = 0.0009) (Figure 7).

#### Disease stage, unknown

Two studies (Su, 2010; Wu et al., 2015) including 116 patients reported no significant difference of MMP-3 between the groups (SMD: −0.3; 95% CI, −0.67 to 0.07; *P* = 0.11).

### Subgroup analyses of the serum MMP-3 by the form of a TCM

#### The form of a TCM, chinese patent drug

**Figure 8:**
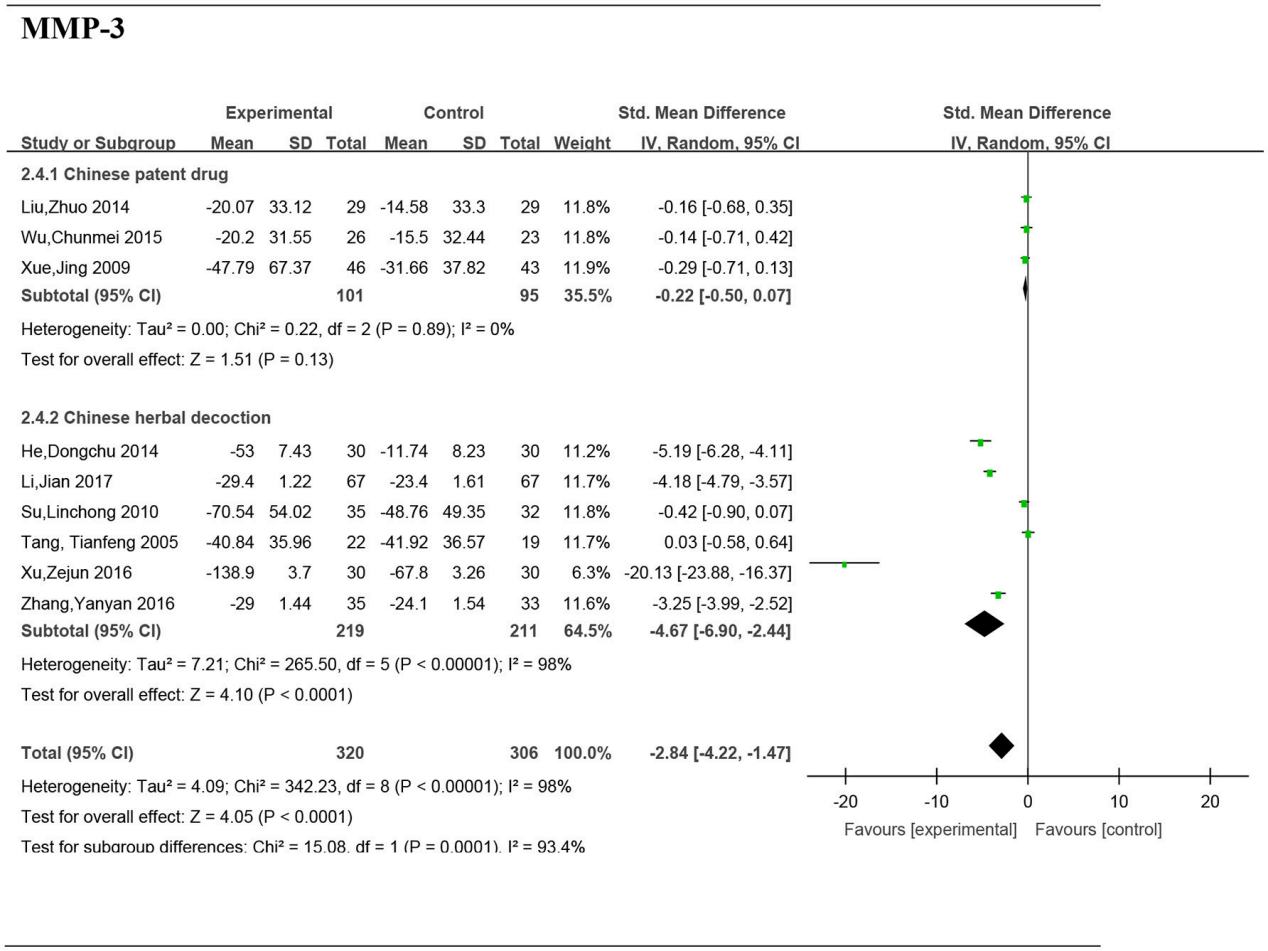
Forest plot and meta-analysis of the serum matrix metalloproteinase 3 of subgroup analysis by the form of a TCM. Experimental: the group of Chinese Medicine; Control: the group of Western medicine; SD, standard deviation; IV, inverse variance method; CI, confidence interval.

Three studies (Xue et al., 2009; Liu, 2014; Wu et al., 2015) including 196 patients reported no significant difference of MMP-3 between the groups (SMD: −0.22; 95% CI, −0.50 to 0.07; *P* = 0.13) (Figure 8).

#### The form of a TCM, chinese herbal decoction

Six studies (Tang et al., 2005; Su, 2010; He and Xiao, 2014; Xu et al., 2016; Zhang et al., 2016; Li et al., 2017) including 430 patients reported a significant difference of MMP-3 between the groups (SMD: −4.67; 95% CI, −6.90 to −2.44; *P* < 0.0001).

### Sensitivity analysis

**Figure 9:**
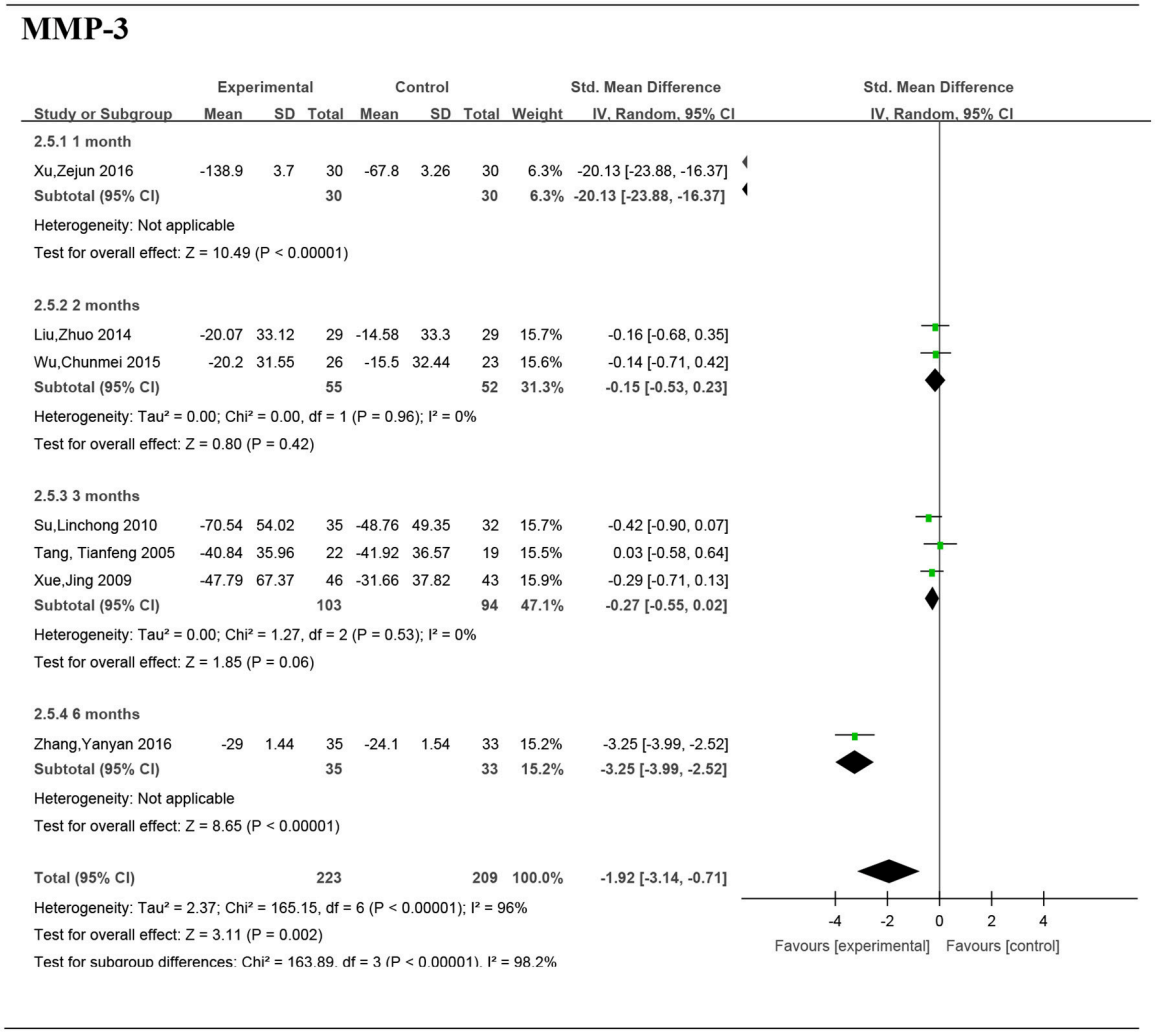
Forest plot and meta-analysis of the serum matrix metalloproteinase 3 of subgroup analysis by intervention duration. Experimental: the group of Chinese Medicine; Control: the group of Western medicine; SD, standard deviation; IV, inverse variance method; CI, confidence interval.

We performed sensitivity analyses to explore the differences in effect size and to assess whether the conclusions were robust for the decision-making process. The studies or trials which were judged as high risk or unclear risk of attrition bias were excluded. There was no change in the significance of any of the outcomes (Table 2). In the subgroup of 3 months treatment, two studies (He and Xiao, 2014; Li et al., 2017) were treated by “dispelling wind and dispersing cold,” while other therapies were to “activate blood circulation and remove stasis,” where both are Chinese medical methods to treat diseases. After excluding these two literatures, there was no heterogeneity in the subgroup, and the overall result did not change (Figure 9).

**Table 2:** Sensitivity analysis.

**Outcomes**	**Studies**	**EG**	**CG**	**MD**		***P*-value**	**Study heterogeneity**			
	**no**.	**no**.	**no**.	**(95% CI)**				**df**	***I^2^*, %**	***P*-value**
**SECONDARY OUTCOMES**										
BMD	5	201	166	0.03	0.08	<0.001	1.3	3	0	0.73
MMP-3	4	199	193	−11.43	−10.5	<0.0001	5090.63	4	100	<0.00001

*Experimental: the group of Chinese Medicines; Control: the group of Western Medicines; CI, confidence interval*.

### Publication bias analysis

Egger's test of BMD did not suggest significant publication bias (*P* = 0.661). There is significant publication bias for MMP-3 (*P* = 0.011) (Table 2).

## Discussion

The aim of this review was to provide an overview of bone-protecting effects of Chinese medicines in the treatment of RA. The review revealed 16 RCTs investigating bone-protecting interventions.

The changes in local metabolism may impair the dynamic balance of bone formation and resorption, finally leading to bone and cartilage destruction of RA (Solomon et al., 2009). As stated, about 90% of the RA patients developed bone erosions within 2 years after onset, eventually processing to joint deformities disability (Miossec, 2013; Nam et al., 2014). Inflammation is the driving force in RA, which gives rise to structural damage during the course of RA (Schett et al., 2008). Conventional DMARDs are extensively used in the treatment of RA, either as monotherapy or in combination with other drugs. Conventional DMARDs have been shown to reduce the expression of the nuclear factor-kappa B ligand in synovial fibroblast cultures, which may indicate a specific effect on the osteoclast (Lee et al., 2004). Among the conventional DMARDs, the most widely used agent is MTX, which is also considered the anchor drug for the treatment of RA. In several evidences, the biologic agents, including the inhibitors of tumor necrosis factor (TNFi), exhibited the effect to somewhat halt the progression of articular erosions (Dohn et al., 2011; Finzel et al., 2011). These agents are usually used in combination with MTX. An RCT comparing denosumab, an antagonist of receptor activator of nuclear factor-kappa B ligand, with placebo in patients of RA showed a statistically significant decrease in erosion score (Cohen et al., 2008). Unfortunately, these biologic agents are too expensive to be widely used in a developing country such as China. Therefore, we urgently need an effective and cheap therapeutic method to block or even treat bone erosion of RA.

We therefore systematically searched and analyzed the available literature to evaluate the efficacy and potential advantages of Chinese Medicine (or combination of Chinese and Western medicine) compared with Western Medicine. In assessing the extent of bone destruction, imaging provides us with a more intuitive evidence. The pooled data of outcomes indicates that Chinese Medicine can improve the imaging findings of RA. Some studies (Ma et al., 2009; Jiang et al., 2012) showed that Chinese medicine has the potential to improve X-ray imaging during RA process. Moreover, the pooled data showed that Chinese medicine (the combination of Chinese and western medicine) are more effective in improving the BMD when compared to the treatment provided using Western medicine only.

Due to the limitation of the number of randomized trials, there is no definite result in the imaging of bone destruction in patients with RA. We try to indirectly reflect the bone protective effect of Chinese and Western Medicine on RA from mineralization of bone tissue, stability of cartilage structure, and degree of bone resorption.

The BMD is an important index reflecting the metabolic status of the skeleton of a human being. The pooled data showed that Chinese medicine (the combination of Chinese and western medicine) is more effective in improving the BMD than only Western medicine treatment.

Pooled data indicated that the MMP-3 level was reduced in the Chinese medicine group. It is known that the MMPs can degrade all the protein components of the cartilage, resulting in destruction of ligaments, cartilage, and bone (Klimiuk et al., 2002). Importantly, MMP-3 has been shown to be mostly related in synovium of advanced RA patients (Klimiuk et al., 2002). Therefore, MMP-3 is defined as a biomarker for bone destruction.

We further carried out a subgroup analysis according to the intervention duration, disease stage, and the form of a TCM, but the heterogeneity in the 3 months test is still very high. Most of the MMP-3 data was tested in the laboratory. In the subgroup of 3 months treatment, two studies were treated by “dispelling wind and dispersing cold,” while other therapies were to “activate blood circulation and remove stasis”; we therefore exclude these two studies. After excluding these studies, there was no significant difference in the subgroup, and the overall result did not change. According to the result, under the intervention of TCM, the serum MMP-3 level could be effectively reduced in a patient with early RA. It is possible that the decoction of Chinese medicine is more effective. The test level difference has a great relationship with the heterogeneity. The precise cause of heterogeneity is unclear, though it may also be due to Traditional Chinese Medicine syndromes and participant characteristics among others.

Pooled data indicated that the Chinese medicine (the combination of Chinese and western medicine) had a better effect in comparison to only Western medicine in reducing the destruction of cartilage.

## Conclusions

This meta-analysis of 16 RCTs or CCTs includes 1,171 patients. Our findings suggest that the Chinese traditional medicine leads to a statistically significant increase in the BMD and decrease in MMP-3, which implies that the Chinese medicine may provide an efficient treatment option for RA in terms of the bone-protecting efficiency, especially to patients in China. However, the limitations of this meta-analysis are also to be noted. All the included studies were performed only in China. Many of the studies included a small number of patients. The most important criteria, including the BMD, imaging stage, and MMP-3, were not reported in all the studies. In addition, the limited numbers of RCTs prevented us from reaching any definitive conclusions. Furthermore, bone destruction is a chronic progression, but the study of the literature for 3–6 months does not give long-term results. Therefore, more studies with high quality are urgently needed to judge the full potential of the Chinese traditional medicine for use in bone-protection. Accordingly, the conclusions of this review should be carefully interpreted. Due to the increasing use of Chinese medicine, accurate and complete data on the interactions between Chinese medicine and western medicine are urgently required.

## Author contributions

XC, X-MC, R-YH, Z-HW, and P-JJ contributed to the literature database search, data collection, data extraction, data analysis, and writing of the manuscript. XX, KB, R-RW, J-HP, H-JL, Q-WY, J-YY, M-JW, HY, J-JL, Y-JH, and Q-CH performed data analysis and rationalization of the results. The topic was conceptualized by P-JJ, R-YH, and Z-HW.

### Conflict of interest statement

The authors declare that the research was conducted in the absence of any commercial or financial relationships that could be construed as a potential conflict of interest.

## Abbreviations

RA: Rheumatoid Arthritis
CNKI: the China National Knowledge Infrastructure
VIP: the VIP Database for Chinese Technical Periodicals
RCTs: randomized controlled trials
EULAR: European League Against Rheumatism
ACR: American College of Rheumatology
SMD: standardized mean difference
MD: mean difference
BMD: bone mineral density
MMP3: Matrix metalloproteinase 3
RR: risk ratio
CIs: confidence intervals
ACR: American Rheumatism Association
OPG: Osteoprotegerin
MMPs: Matrix metallo proteinases
TCM: traditional Chinese medicine.
